# An investigation into the shifting landscape preferences of rural residents in Taiwan and their relationship with ecological indicators

**DOI:** 10.1038/s41598-024-77045-x

**Published:** 2024-11-13

**Authors:** Fuer Ning, Hui Wang, Yu-Chen Chien, Haozhang Pan, Sheng-Jung Ou

**Affiliations:** 1 College of Art Design/College of Landscape Architecture, Nanjing Forestry University, No 159, Longpan Rd., Xuanwu District, Nanjing, China; 2 College of Landscape Architecture, Nanjing Forestry University, No 159, Longpan Rd., Xuanwu District, Nanjing, China; 3 Doctoral in Architecture and Urban Design Program, Department of Architecture, College of Design, Chaoyang University of Technology, Taichung, 413 Taiwan; 4 Department of Landscape and Urban Design, College of Design, Chaoyang University of Technology, Taichung, Taiwan

**Keywords:** Rural community, Landscape change, Landscape ecological indicators, Landscape preference, Taiwan, Human behaviour, Psychology and behaviour, Sustainability

## Abstract

Rapid urbanization has significantly altered landscape environments in both urban and rural regions, and these landscapes have been demonstrated to play a pivotal role in human well-being. This study develops a coherent framework that integrates landscape change, landscape ecological indicators, and landscape preferences within the context of the evolving landscape environments of rural communities in Taiwan. Four distinct types of rural communities were selected, and a quantitative methodology was employed to investigate the variations and transformations in landscape preferences among rural residents in the context of landscape change. A qualitative methodology was employed to investigate the relationship between landscape ecological indicators and landscape preferences. The study’s findings indicate significant temporal variations in residents’ landscape preferences, with landscape beauty, stewardship, and coherence emerging as key determinants in the evaluation of these preferences. Landscape ecological indicators were found to be significantly correlated with variables such as landscape complexity, landscape beauty, openness, naturalness, and comprehensive landscape assessment. The findings of this study indicate that design planners, land managers, and public sector organizations can employ multidimensional thinking in the management of rural landscapes to align with the visual aesthetic preferences of rural residents.

## Introduction

In 2005, the Millennium Ecosystem Assessment was the pioneering comprehensive evaluation of the landscape environment’s contribution to human well-being and received widespread recognition. The relationship between human well-being and the landscape environment is gaining increasing recognition within scientific research, policy formulation, and environmental management practices^1^. Therefore, to enhance and ensure human well-being, it is imperative to consider the implications of landscape change. Climate change and urbanization have resulted in significant alterations to landscapes, affecting both urban and rural regions^2,3^.

Rural regions are imbued with significant economic, socio-cultural, industrial, and ecological significance^4–7^. Agricultural and rural policies have instigated transformations within the agricultural industry system—specifically intensification, scale expansion, and increased agricultural waste—alongside shifts in employment patterns and lifestyles. These changes have, in turn, led to alterations in the rural landscape environment^8–10^. In Europe, initiatives such as the Common Agricultural Policy (CAP) subsidies have incentivized farmers to implement environmentally sustainable agricultural practices. This has mitigated the adverse environmental impacts of farming, enhanced the diversity of agricultural landscapes, and improved ecological connectivity^11,12^. Changes in the rural landscape environment are evidenced by alterations in land use patterns and ecological conditions^13^, as well as variations in the visual quality of the landscape^10^. Thus, landscape change is manifested not only at the objective level, as evidenced by physical landscape indicators, but also at the subjective level, as reflected in landscape preference assessments^14^. By integrating the subjective and objective dimensions of the study, the landscape environment can become more attuned to the needs of residents, thereby enhancing their life satisfaction. This approach also enables policymakers to adapt and develop landscape management policies in response to evolving conditions^15^.

A reciprocal relationship between changes in the landscape and landscape preferences, deemed pivotal for the formulation of landscape policy^16^. The profound impact of transformations in the landscape, encompassing facets such as land use type, intensity, and management, on human preferences has been extensively acknowledged^8,17–19^. Landscape preferences are the product of the interaction between the physical characteristics of a landscape and the psychological attributes of the observer^20^. It is the result of multisensory input^21,22^. It is the response generated by human beings when faced with various types of landscapes, which is centred on the degree of preference for the landscape^18^. Changes in landscape preferences contribute to landscape change. Human perceptions of the landscape have changed over time, placing greater emphasis on landscape quality, which in turn has changed the landscape environment^23^. Arnberger and Eder^8^ delved into preference heterogeneity by scrutinizing historical landscape environment change processes in terraced areas. Foley^17^ conducted a case study on attitudes towards rural landscape settlements and shifts in agricultural practices, utilizing the Irish rural landscape as a case study. Xu et al.^19^ investigated alterations in landscape preferences amid urbanization in Fujian Province. Ning and Ou^18^ examined shifts in people’s landscape preferences over time by analyzing landscape environment changes in an urban fringe area. However, the extent to which these findings can be adapted to rural communities in Taiwan is controversial. Landscape preferences evolve in response to shifts in social norms and expectations, as well as alterations in the landscape itself^24^. The development of Taiwan’s rural communities and the formulation of policies are quite different from those in Europe and the United States. Taiwan’s rural community policy began in 1973 with a shift in emphasis from agricultural production to rural life. It shifted to the integrated development of life, culture, production and ecology, and then to the integrated development of life, production, ecology and the future. In summary, exploring residents’ landscape preference factors and landscape change evaluation in the context of landscape change in rural communities in Taiwan has not been adequately researched.

Changes in the landscape environment are often monitored and analyzed through the utilization of landscape ecological indicators^25,26^. The fundamental component of the landscape resides in its land cover structure. Landscape ecological indicators function as a digital representation of this structure^27^. The examination of changes in the landscape environment entails the digitization of the land cover structure, a methodological approach applicable not only to urban and fringe areas for the analysis of landscape environmental changes^28–31^, but also extends to rural areas^9,32,33^. Wang and Wen^32^ employed landscape ecological indicators, including the percentage of landscape (PLAND), Shannon diversity index (SHDI), patch density (PD), and edge density (ED), in their investigation of changes in the landscape environment within traditional villages in the Enshi forest area of China. Geng et al.^34^ employ five landscape indicators—total area (TA), patch number (NP), average patch size (MPS), fragmentation index (FN), and patch density (PD)—to quantify the level of dispersion and fragmentation. The results of the study show that there are differences in the changes in the spatial patterns of rural communities along roads of different grades. Rural communities near national roads are the most compactly distributed, while those along provincial roads are the densest. Sun et al.^35^ employed metrics such as total class area (CA), number of patches (NP), mean patch size (MPS), and patch size standard deviation (PSSD) to examine changes in the landscape environment within the transition zone between mountains and plains.

Landscape preferences are correlated with landscape ecological indicators, and studies that combine the two are subjective and objective in unity^27^. Crawford^36^ and Palmer^37^ recognize landscape preferences as a response to landscape ecological attributes. For example, patch size and edge density can account for naturalness. Molnarova et al.^38^ found that farmland patch size and heterogeneity had an effect on landscape preferences. Palmer^24^ found that some landscape ecological indicators explain landscape preferences and some do not. Uninterpretable because of the complexity of the landscape itself, which is affected by landscape type classification, landscape granularity, landscape visibility, sample size, scope, and so on. Therefore, there are differences in the relationship between landscape ecological indicators and landscape preferences. This part of the study will help to improve the interpretation of ecological indicators and help people to understand the impacts on preferences through landscape ecological changes^15^.

**Fig. 1:**
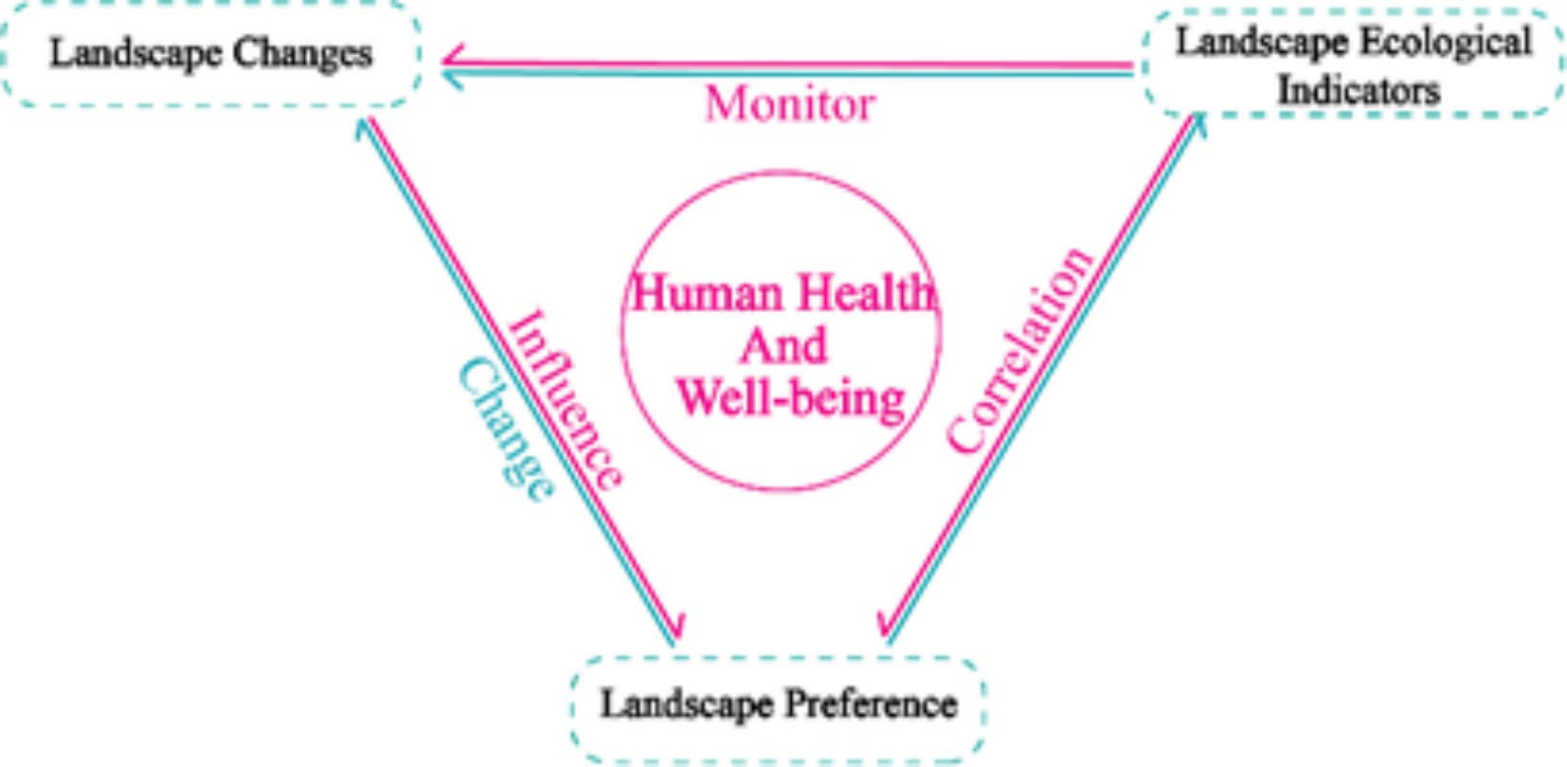
A conceptual framework for the interconnection of landscape change, landscape ecological indicators and landscape preferences.

This research establishes a logical framework centered on the interplay among changes in the landscape environment, landscape ecological indices, and preferences for the landscape (Fig. 1). The research encompasses the time frame from 2007 to 2022, incorporating survey data on alterations in landscapes, ecological indicators, and preferences gathered from diverse rural communities across Taiwan throughout a period of 15 years. The principal aims involve investigating: (1) the fluctuations and discrepancies in landscape preferences among rural community residents across distinct time intervals, and identifying the factors that influence landscape evaluation during each of these two periods, and (2) the correlation between indicators of landscape ecology and preferences for landscapes.

## Materials and methods

### Study area and scope of research

Taiwan’s rural communities are divided into three major types and four categories. The three types include living type, production type and ecological type communities. Living type communities combine agricultural production and residents’ lives, emphasising sustainable development, community participation and ecological protection. Through the provision of amenities and cultural activities, it enhances the quality of life of rural residents and community cohesion. Production type communities are based on land management, agricultural production, food supply, and the production and sale of economically valuable crops to make a living for the community. Ecological type communities are clearly orientated towards the ecological features of the environment, and are rich in ecological resources, with a view to increasing economic income through the tertiary sector. The four categories of rural communities are based on the principles of living, production and ecological space, and the ratio of industrial production and development willingness^39^, resulting in the holistically prosperous and beautiful community, rural lifestyle experience community, economic production community and ecological conservation community (Table 1). The Holistically Prosperous and Beautiful community has an equal distribution of space and occupancy for living, production, and ecological functions. The Rural Lifestyle Experience community is based on the function of living, supplemented by ecological function. Although the production function is still based on primary and secondary industries, it is not the mainstay of the economic resources of the residents of the rural community. The community environment and facilities have reached a relatively high standard, and with the spare capacity, the preservation and maintenance of the natural environment and culture of the villages has become an important direction. The Economic Production community is based on agricultural production, and the production function of primary and secondary industrial development is larger than the living function, and ecological space is not the main development. The proportion of the Ecological Conservation Community’s ecological function is much larger than the production function and living function, although the community has a small proportion of primary and secondary industrial production. However, as the community is rich in ecological resources, the development of homestay experience and eco-education will expand the income.

**Table 1:** Classification of rural communities in Taiwan.

Three major types	Four categories	Proportional distribution
Living type	Holistically Prosperous and Beautiful community	Living ≥ Production ≥ Ecological
	Rural Lifestyle Experience community	Living ≥ Ecological > Production
Production type	Economic Production community	Production ≥ Living > Ecological
Ecological type	Ecological Conservation community	Ecological > Production ≥ Living

The study area was determined by cross-checking the top ten Classic Farming and Fishing Villages with the winning areas of the Gold Medal Rural Community Competition (Table 2). The selection of the top ten Classic Farming and Fishing Villages is a selection activity launched to construct a new realm of agriculture in Taiwan, to gain the encouragement and support of the general public, and to stimulate the income of the fishermen^40^. It is expected to demonstrate the charm of Classic Farming and Fishing Villages in terms of production, living, and ecology. The Gold Medal Rural Community Competition draws on the successful experience of the German Rural Competition and establishes a selection mechanism that is in line with Taiwan’s rural development^41^. Rural communities are encouraged to show their uniqueness, innovative projects, and sustainable future.

**Table 2:** Selection of study areas.

	Production type	Living type	Ecological type
Holistically Prosperous and Beautiful community		Huashan community (Yunlin county); Fuzhi community (Miaoli county); Seshui community^✮^; Zhaomen community (Xinzhu county)	
Economic Production community	Dalian community^✮^	Wuhe community (Hualian county)	
Ecological Conservation community			Taian community (Hualian county); Gangbian community^✮^
Rural Lifestyle Experience community		Shuangtan community*^✮^; Yongan community* (Taidong county)	

Not labelled as Classic Farming and Fishing Villages; *Gold Medal Rural Communities; ^✮^Study Areas.

In addition, the study area was selected with reference to the results of the Land Use Survey to identify rural communities with significant landscape changes. At the same time, it ensures that the selected rural communities have different orientations and categories. Finally, the production type was determined to be the Dalian community (Economic Production community) in Tianwei Township, Changhua County. Living type communities are Seshui community and Shuangtan community. Seshui community is a Holistically Prosperous and Beautiful community, located in Dayan Village, Yuchi Township, Nantou County. Shuangtan community is a Rural Lifestyle Experience community, located in Shuangtan Village, Sanyi Township. Ecological type is Gangbian community (Ecological Conservation community) in Suao Township, Yilan County.

**Fig. 2:**
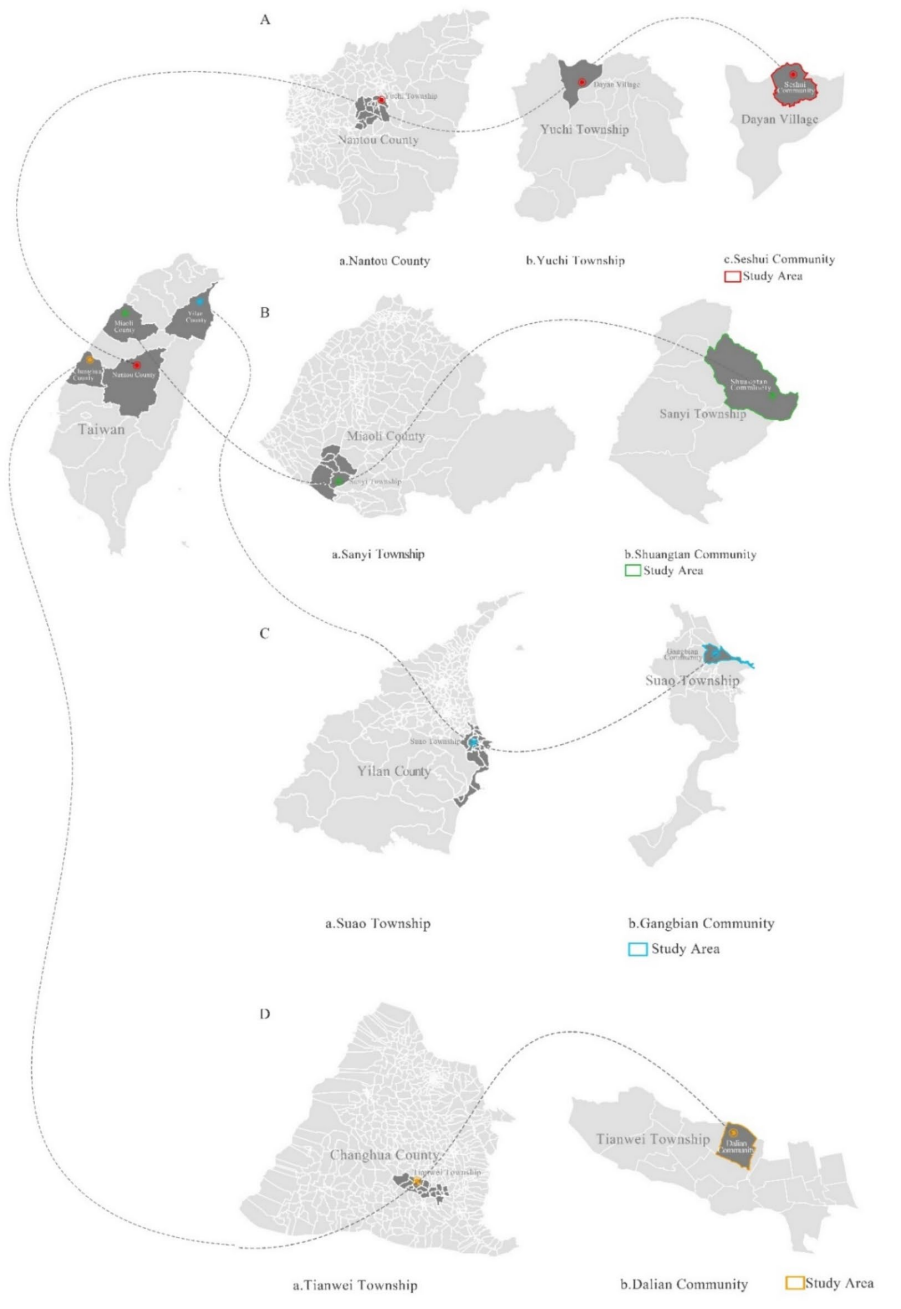
District analysis^9^.

(1) Seshui community (Fig. 2A) is located along Taiwan Highway 21, surrounded by mountains, with terrain like a lotus flower valley, at an altitude of about 600 to 800 m above sea level. The total area of the community is 110.18 ha. There are 286 households (688 people) in the community^42^. The community offers the opportunity to experience scenic views and special features such as the Rural Trail, Waterfalls, Mystic Valley, Houpigou Wetland, Jintian Temple, Tujiao Alley, and the Double Heart Ecological Pond. Pottery and black tea are the speciality products of the community.

(2) Shuangtan Community (Fig. 2B) is located in the hilly area of Guanjiaoshan Mountain, 700 m above sea level, with County Road 130 running through it. The total area of the community is 1326.94 ha. At present, there are about 180 households (445 people) in the community^43^, which is dominated by the Hakka ethnic group. Residents of the community are mainly engaged in wood carving, tourism and farming. The community is home to the historic Shengxing Railway Station, Hakka cuisine and a variety of colorful paintings. The community focuses on the revitalisation of old settlements and the revitalisation of new and old industries. Promote the forest industry, and ecological sustainable development of the happy settlement and craft village, in order to improve people’s lives.

(3) Dalian Community (Fig. 2C) is located in the western plain next to Provincial Highway 1 and was named after the sickles made by the ancestors who opened up the wasteland. The total area of the community is 143.95 ha. There are 450 households (1950 people) in the community^44^. Its history of floriculture dates back to the Qing Dynasty. Its industry was already sizable during the days of the Japanese occupation, setting the stage for the growth of the community’s floral industry. The community offers a wide range of activities and features such as bike rides, nighttime chrysanthemum tours and a flower auction center.

(4) Gangbian Community (Fig. 2D) is located in the southernmost part of the Lanyang Plain and borders the northernmost part of the Central Range, a cluster with a coastline. The total area of the community is 299.37 ha. There are 518 households (1395 people) in the community^45^. In 2001, the community introduced “eco-communities” as the main axis of development in order to develop a sustainable community. The community has 102 hectares of important wetlands recognized by the International Union for the Conservation of Wetlands (IUCN) and is a dry port bird sanctuary inhabited by more than 170 species of birds. In recent years, the community has been putting forward revitalisation projects such as the “Green Flexible Infinite Harbour”^46^.

### Research framework and hypotheses

**Fig. 3:**
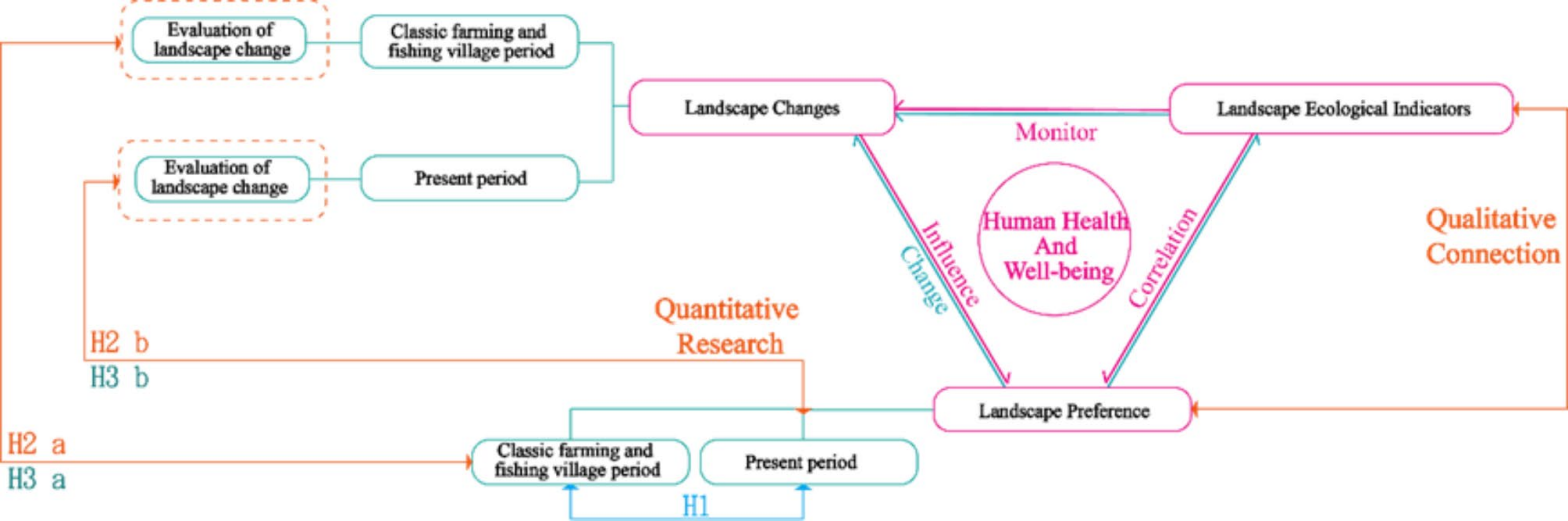
Research framework and hypotheses.

Firstly, a conceptual framework of interconnections between landscape change, landscape ecological indicators, and landscape preferences was constructed (Fig. 1). Secondly, the literature review method was used to find the measurement variables of landscape preference factor and landscape change evaluation. A framework for correlating landscape preference factors with landscape ecological indicators was constructed (Table 3). Next, the relationship between changes in the landscape environment and landscape preferences is discussed using a quantitative research approach. Finally, a qualitative study was conducted to verify the relationship between landscape ecological indicators and landscape preferences (Fig. 3).

**Table 3:** Correlation table between landscape preference and landscape ecological indicators.

Serial number	Landscape preference	Related ecological indicators	Positive/negative correlation	Sources
1	Mystery	AREA	+	Chung^60^
2	Openness	AREA	−	Chung^60^
		FRAC_AM	+	Chung^60^
		PLAND	None	Dramstad et al.^15^
				Fry,et al.^61^
		ENN_MN	None	Fry et al.^61^
3	Complexity	AREA	None	Fry et al.^61^
		SIEI	None	Gonzalo et al.^62^
				Fry et al.^61^
		CONTAG	None	Gonzalo et al.^62^
		ED	None	Fry et al.^61^
		DFLD	None	Gonzalo et al.^62^
4	Landscape Beauty	SIEI	+	Gonzalo et al.^62^
		SIDI	+	Gonzalo et al.^62^
				Schirpke et al.^63^
		SHDI	+	Schirpke et al.^63^
		CONTAG	−	
		FRAC_AM	+	
		SHAPE_AM	+	
		MSIEI	+/−	Schirpke et al.^63^
				Schirpke et al.^63^
		AREA_MN	+	Schirpke et al.^64^
		SHAPE_CV	+	
		Core_MN	None	
5	Landscape Preference	AREA	+	Dramstad et al.^15^
		NP	+	
		TE	+	
		SHEI	+	
		PD	None	
6	Legibility	SIEI	−	Schirpke et al.^64^
		SIDI	−	Dramstad et al.^15^
		NP	−	
7	Landscape Value	ED	+	Palmer^24^
8	Naturalness	FRAC_AM	None	Fry et al.^61^
		PLAND		
9	Coherence	PLAND	None	Fry et al.^61^

The research hypotheses exploring the relationship between landscape preference factors and landscape change evaluation are as follows:

*Hypothesis 1(H1): There were significant differences in landscape preference factors between Classic Farming and Fishing Villages and the present period.*

*Hypothesis 2 (H2)* *:* *There were significant correlations between landscape preferences and evaluation of landscape change in Classic Farming and Fishing Villages and in the present period.*

- H2-a: The landscape preference related factors for Classic Farming and Fishing Villages was correlated with the evaluation of landscape change.
- H2-b: There is a correlation between the factors related to landscape preference and the evaluation of landscape change in the present period.

*Hypothesis 3 (H3): There is a linear relationship between landscape preference related factors and the evaluation of landscape change.*

- H3-a: The factors related to landscape preference during the Classic Farming and Fishing Villages period showed a linear relationship with the evaluation of landscape change.
- H3-b: There is a linear relationship between factors related to landscape preference and the evaluation of landscape changes in the present.

#### Selection and determination of landscape preference related factors and landscape change evaluation measurement variables

Preferences for landscapes are subject to change over time^47^. The factors affecting landscape preferences can be generally categorized into two groups: evolutionary theories and cultural preference theories^48^. The prospect/refuge theory is encompassed within evolutionary theory^49^. Wilson^50^ proposed biophilia. Kaplan and Kaplan^51^ developed the theory of landscape preference within the framework of the landscape perception model. This theory encompasses constructs such as coherence, legibility, complexity, and mystery. Tveit et al.^52^ condensed nine fundamental conceptual frameworks concerning the visual landscape, encompassing stewardship, coherence, disturbance, historicity, visual scale, imageability, complexity, naturalness, and ephemera. Hunter and Askarinejad^53^ integrated theories associated with landscape to formulate ten constructs: naturalness, structure coherence, structural form, depth cues, openness, information gathering support, access, safety, and engagement. Ning and Ou^18^ employed qualitative research to investigate the preferences of the public in urban fringe areas concerning the landscape. These preferences were subsequently categorized into three groups with ten dimensions. The natural factors encompassed elements such as naturalness, reduced pollution, a flat skyline, and organic agricultural production. Human factors included considerations like high accessibility, environmental management, and living convenience. Space perception comprised aspects such as place attachment, landscape beauty, and a sense of landscape experience. The cultural preference theory posits that variations in individual characteristics such as age, gender, occupation, experiential background, educational history, length of residence, and others play a significant role in shaping preferences for landscapes^2,15,19,54–56^. Variations in landscapes, categories, and components influence individuals’ preferences for landscapes^19,57,58^.

In this research, the investigation is founded upon a selection of ten factors (Table 4), namely complexity, coherence, mystery, management, disturbance, openness, naturalness, distinctiveness, fascination, and landscape beauty. Table 1 illustrates the determination of these landscape preference factors.

**Table 4:** Landscape preference factor determination.

No.	Factors	Definition	Source
1	Complexity	Spatial landscape elements are diverse and rich in character, deriving their identity from a variety of forms, shapes, colors, and textures	Kaplan and Kaplan^51^, Tveit et al.^52^, Hunter and Askarinejad^53^
2	Coherence	There is visual unity through the aggregation of patterned landscape elements, colors, and connections	Kaplan and Kaplan^51^, Tveit et al.^52^, Hunter and Askarinejad^53^
3	Mystery	The landscape allows the viewer to explore and imagine	Kaplan and Kaplan^51^
4	Stewardship	Has a sense of order and care, reflecting a sense of human concern through active and careful management	Tveit et al.^52^, Ning and Ou^18^
5	Disturbance	Species invasion, alteration, lack of contextual adaptation, and coherence	Tveit et al.^52^, Ning and Ou^18^
6	Openness	Landscape-scale, visibility, and perception of openness	Appleton^49^, Tveit et al.^52^, Hunter and Askarinejad^53^, Ning and Ou^18^
7	Naturalness	Any landscape type with natural ingredients, or close to the state of nature	Wilson^50^, Tveit et al.^52^, Hunter and Askarinejad^53^, Ning and Ou^18^
8	Distinctiveness	Have the spirit and character of the place, etc	Tveit et al.^52^
9	Fascination/Engagement	Based on the existence of physical things that attract attention	Kaplan and Kaplan^51^, Hunter and Askarinejad^53^
10	Landscape beauty	The landscape has visual beauty, meaning "beautiful, good-looking"	Wartmann et al.^98^^,^^16^, Ning and Ou^18^

By organizing and assessing, a set of ten measurement variables for the landscape preference factor was formulated, and redundant variables were excluded from the ultimate rating scale (Table 5).

**Table 5:** Measurement variables of landscape preferences.

No.	Factors	Factor description	Source	Measurement standards
1	Complexity	The landscape in my area is very varied	Wartmann et al.^16^	Five-point Likert scale 1 = Strongly disagree; 2 = Disagree; 3 = Common; 4 = Agree; 5 = Strongly agree
		The landscape arrangement in my area is complex		
2	Coherence	The landscape in my area is generally harmonious	Referring to Kuo^99^, this study develops	
		There is a sense of continuity and continuity in the landscape of my area	Referring to Ning and Ou^71^, this study develops	
3	Mystery	The landscape in my area is mysterious	Kienast et al.^100^	
		The landscape in my area deserves to be explored and discovered		
		I would like to know more about the landscape in my area		
4	Stewardship	The landscape in my area is well-maintained and managed	Referring to Kuo^99^; Ning and Ou^71^, this study develops	
		My area can manage problematic environments	Referring to Ning and Ou^71^, this study develops	
5	Disturbance	The natural scenery and man-made construction in my area are in harmony	Referring to Kuo^99^, this study develops	
		There is less pollution in my area	Referring to Ning and Ou^71^, this study develops	
6	Openness (Visual Scale)	The views in my area are vast	Referring to Tveit et al.^52^, this study develops	
		The view in the area where I am is not affected by man-made construction and the view is wide	Referring to Kuo^99^, this study develops	
7	Naturalness	The natural environment in my area is good	Referring to Fry et al.^61^, Tveit et al.^52^, this study develops	
		The environment in my area is less man-made		
		There is a lot of wildlife in my area		
8	Distinctiveness	The landscape in my area is unique	Wartmann et al.^16^	
		The landscape of my area adds to the uniqueness of the area		
9	Fascination	The landscape in my area is stunning	Kienast et al.^100^	
		Many things grab my attention in the landscape of my area	Kienast et al.^100^; Wartmann et al.^16^	
		Many places in my area make me want to stay longer	Wartmann et al.^16^	
10	Landscape Beauty	The landscape in my area is very beautiful	Wartmann et al.^16,98^	
		I love the landscape in my area		
		The farmland and growing areas in my area provide stress relief and are a landscape to appreciate	Referring to Ning and Ou^71^, this study develops	

The most intuitive response to landscape changes is changes at the physical level. Changes at the physical level will directly affect people’s evaluation of landscape changes^59^. A literature review was used to develop measurement variables for the evaluation of landscape change in rural communities, and to explore the relationship between factors related to landscape preference and the evaluation of landscape change (Table 6).

**Table 6:** Measurement variables for landscape change.

Factors	Factor Description	Source	Measurement Standards
Landscape Change Evaluation (Two periods)	Overall, I give a high score to the landscape preference of the 2007 classic farming and fishing village period	Referring to Ning and Ou^99^, this study develops	Decile scale 1 = Strongly disagree; 10 = Strongly agree
	Overall, I give a high score to my preference for landscapes in the present period		

#### Selection and determination of landscape ecological indicators

The correlation between landscape ecological indicators and landscape preferences is shown in Table 7. Mystery has a positive correlation with AREA^60^. Chung^60^, Fry et al.^61^, and Dramstad et al.^15^ confirmed that openness is negatively correlated with AREA and positively correlated with FRAC. Landscape beauty is positively correlated with SIEI/SIDI/SHAPE_AM/FRAC_AM/AREA_MN/SHAPE_CV/ SHDI, negatively correlated with CONTAG, and the relationship with CORE_MN/MSIEI is of uncertainty^62–63^. Dramstad et al.^15^ found a positive correlation between landscape preference and AREA/NP/TE/SHEI. Legibility is negatively correlated with SIEI/SIDI/NP^61,64^. Palmer^24^ found a positive correlation between landscape value and ED. To ensure the comprehensiveness of landscape ecology indicators, eight distinct dimensions were integrated. These dimensions include Area Index, Density Magnitude and Variation, Edge Index, Shape Index, Core Area, Proximity, Diversity Index, and Spread Index. The selection of specific landscape ecological indicators, namely AREA, PLAND, NP, PD, TE, ED, FRAC_AM, SHAPE_AM, Core_MN, ENN_MN, SHDI, SHEI, SIDI, SIEI, and CONTAG, resulted from a meticulous filtration process.

**Table 7:** Sample analysis.

Basic information		Number	Percentage	Basic information		Number	Percentage
Survey the community	Seshui Community	30	20.2%	Age	Under 40	29	19.6%
	Dalian Community	47	31.8%				
					41–50	25	16.9%
	Gangbian Community	32	21.6%				
	Shuangtan Community	39	26.4%		51–60	42	28.4%
Gender	Male	74	50.0%		61–70	22	14.9%
	Female	74	50.0%		Over 71	30	20.2%
Education level	High school and below	76	51.3%	Environmental work	Yes	33	22.3%
	College or university	58	39.2%		No	115	77.7%
	Master degree and above	14	9.5%	Living Environment	Urban	12	8.1%
Length of residence	11–20	39	26.4%		Suburbs	26	17.6%
	21–30	30	20.3%		Rural	110	74.3%
	More than 30 years	79	53.3%	Total		148	100.0%

### A quantitative study of landscape preference related factors and evaluation of landscape change

**Fig. 4:**
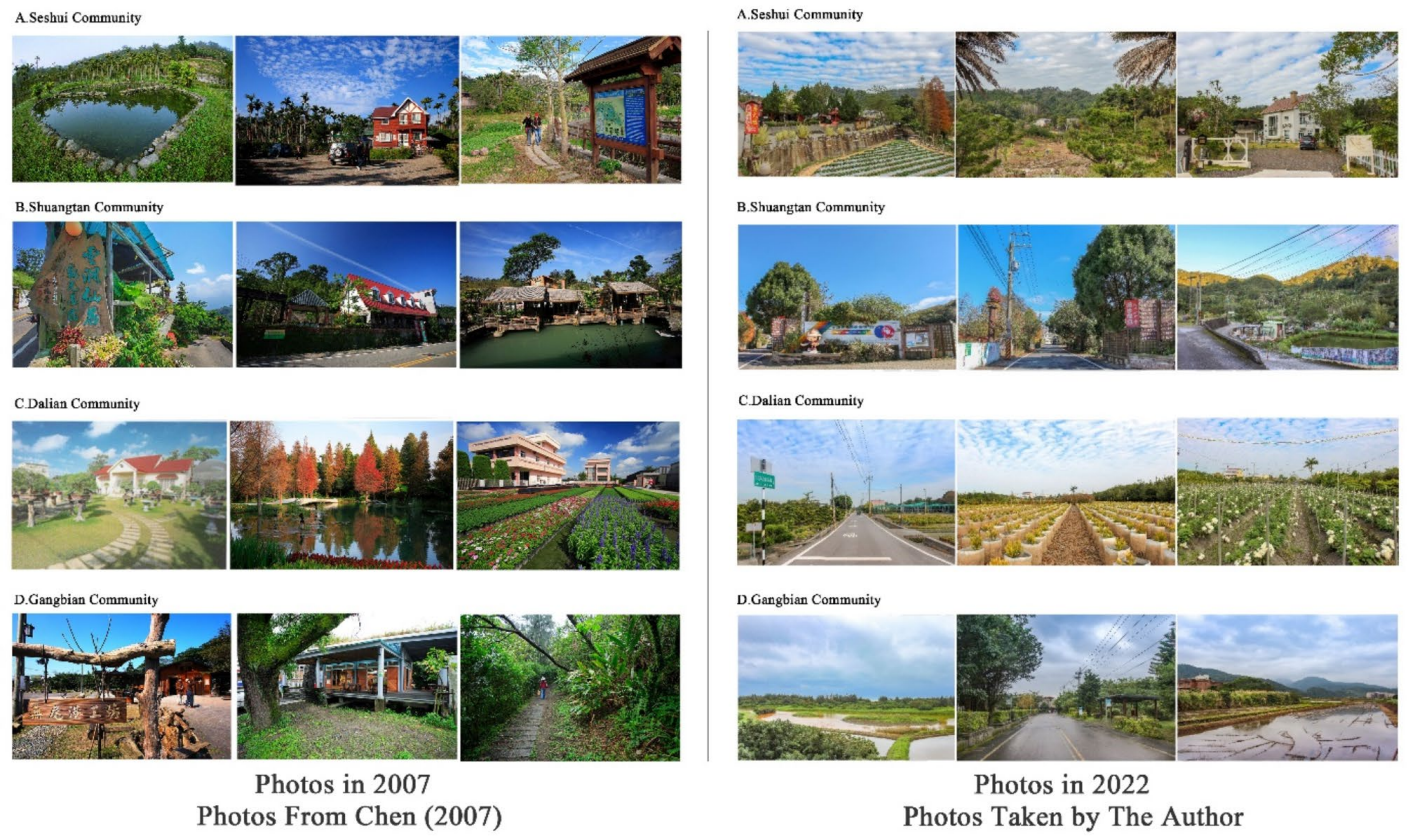
Photos of four rural communities in 2007 and 2022. Photos from Chen^40^ and the author.

A questionnaire survey was used for data collection to investigate the relationship between landscape preference related factors and the evaluation of landscape change. The base area is based on a simplified version of the rural regeneration areas published in 2018. To ensure the reliability of the questionnaire. Firstly, at the questionnaire design stage. The variables measured in the questionnaire were derived from the use and collation of journal literature, as described in detail in “Selection and determination of landscape preference related factors and landscape change evaluation measurement variables”. In addition, three experts were invited to conduct a pre-test of the questionnaire to verify the linguistic description and appropriateness of the questionnaire. Secondly, at the questionnaire stage, the questionnaire survey used an intention sampling method, and respondents were required to have lived in the study area since 2007. Prior to the official start of the survey, respondents were identified as target respondents by showing them photographs of Classic Farming and Fishing Villages 2007 (Fig. 4) and a short semi-structured interview. Photographs^40^ from the Agency of Rural Development and Soil and Water Conservation, Ministry of Agriculture, were screened to remove photographs of people and to focus on environmental photographs. The researcher will need to confirm the location of the environmental sites in the photographs through fieldwork to ensure that the selected photographs are located within the community boundaries. The photographs are combined with semi-structured interviews centred around, are you familiar with the landscapes in the photographs? Do you remember the Classic Farming and Fishing Villages period community? If so, do you prefer Classic Farming and Fishing Villages or present period landscapes? Why? If you have no impression, do not fill in the questionnaire. Finally, the target subjects filled out the questionnaire. The questionnaire was completed through community photographs and short semi-structured interviews (Fig. 4) that elicited memories of past environments from the locals. Data collection was completed between 17 March and 19 April 2022. At least 30 valid questionnaires were collected from each community, which is statistically considered a large sample. The reliability and validity of the questionnaires were examined by SPSS to ensure their reliability.

The analysis of the relationship between the evaluation of landscape changes and preferences was conducted through the utilization of SPSS 26.0 (https://www.ibm.com/support/pages/spss-statistics-260-fix-pack-1). Paired-sample *t*-tests, Pearson’s correlation analyses, and multiple regressions were employed for this purpose. Hypothesis 1 employed paired sample *t*-tests to assess the differences in landscape preferences between the Classic Farming and Fishing Villages and the present period. This method evaluates the difference within the same cases during both the pre- and post-periods. Hypothesis 2 posited that the examination of the relationship between the evaluation of landscape changes and landscape preferences would involve the application of Pearson’s correlation analysis. This method is utilized to ascertain the extent of a relationship between two random variables. The third research hypothesis was to investigate the multiple linear regression analysis of landscape preference related factors and evaluation of landscape changes. Employing this method involves examining the correlation between two or more phenomena and can yield a predictive model.

### Qualitative association of landscape ecological indicators with landscape preference related factors and evaluation of landscape change

The data for the landscape ecological indicators are (objectively) derived from the quantification of the geographic space of rural communities. Firstly, the classification of land use in rural communities, through a practical survey of rural communities, the types of land use are classified into six categories: farmland, forest land, transport land, water body land, construction land and other land. Secondly, ArcGIS 10.5 (https://desktop.arcgis.com/zh-cn/quick-start-guides/10.5/arcgis-desktop-quick-start-guide.htm) was used to produce spatial patterns of the landscape to show the land use more clearly. Next, Fragstats 4.2 (https://fragstats.software.informer.com/4.2/) was used to calculate the landscape ecological indicators that were selected to be associated with landscape preference factors. Landscape preference related factors and evaluation of landscape change data (subjective) are derived from the questionnaire survey. Each landscape preference related factors and evaluation of landscape change data was calculated as an average of one and more variables. Finally, the qualitative association of landscape ecological indicators with landscape preference related factors and evaluation of landscape change was explored. There is a need to map trends in landscape ecological indicators, landscape preferences, and evaluation of landscape change. The trend analysis graphs were compared and analyzed to obtain the preliminary findings. The correlation of the indicators is then verified by combining the support of existing literature.

## Results

### Sample analysis

A total of 148 valid samples were collected in the rural community survey, with the Seshui community accounting for 20.20% (30 samples), the Shuangtan community 26.40% (39 samples), the Dalian community 31.80% (47 samples), and the Gangbian community 21.60% (32 samples). Respondents were evenly distributed in terms of gender. The majority of the respondents were over 40 years of age. More than half of the respondents had a high school education level. The proportion of people who have not worked in the environment-related field is even higher. The number of people who have lived in the city for more than 30 years is relatively high (Table 6).

### Differences in landscape preference related factors between classic farming and fishing villages and present period

According to the findings presented in Table 8, it is evident that the research hypothesis H1 holds true, indicating a noteworthy contrast in residents’ landscape preferences (specifically regarding complexity, coherence, stewardship, naturalness, fascination, and landscape beauty) between Classic Farming and Fishing Villages and present period of the rural community. The statistical significance of these differences is consistently below the threshold of 0.05. Except for naturalness, the ratings for all landscape preferences are notably increased in the present period. During the period of Classic Farming and Fishing Villages, the natural setting exhibited greater favorability, characterized by fewer human-made structures and an abundance of wildlife.

**Table 8:** Difference in landscape preference related factors between 2007 classic farming and fishing villages and present period in rural communities.

Title	Time	Mean	S.D	T	Sig
1. Complexity	2007	3.22	0.866	− 5.432	0.000***
	Present	3.67	0.808		
2. Coherence	2007	3.64	0.920	− 2.378	0.019*
	Present	3.86	0.956		
3. Mystery	2007	3.57	0.799	− 1.701	0.091
	Present	3.71	0.876		
4. Stewardship	2007	3.43	0.856	− 4.047	0.000***
	Present	3.82	0.960		
5. Disturbance	2007	2.25	0.770	− 0.427	0.670
	Present	2.29	0.888		
6. Openness	2007	3.86	0.881	1.162	0.247
	Present	3.75	1.019		
7. Naturalness	2007	3.87	0.882	4.863	0.000***
	Present	3.49	0.912		
8. Distinctiveness	2007	3.69	0.904	− 1.921	0.057
	Present	3.84	0.935		
9. Fascination	2007	3.58	0.864	− 3.517	0.001**
	Present	3.85	0.889		
10. Landscape beauty	2007	3.83	0.801	− 2.972	0.003**
	Present	4.05	0.854		

P < 0.001***, P < 0.01**, P < 0.05*

### Correlation analysis between landscape preference related factors and evaluation of landscape change

#### The landscape preference related factors for classic farming and fishing villages was correlated with evaluation of landscape change

As depicted in Table 9, the research hypothesis H2-a is upheld, demonstrating noteworthy correlations between the evaluation of landscape change during the period of Classic Farming and Fishing Villages and landscape preference related factors, excluding complexity. Disturbance exhibits a negative correlation, whereas coherence, mystery, stewardship, openness, naturalness, distinctiveness, fascination, and landscape beauty demonstrate positive correlations.

**Table 9:** The correlation between evaluation of landscape change and landscape preference related factors in the Classic Farming and Fishing Villages period.

Landscape preference related factors	Evaluation of landscape change
b1 Complexity	0.051
b2 Coherence	0.371**
b3 Mystery	0.379**
b4 Stewardship	0.371**
b5 Disturbance	− 0.418**
b6 Openness	0.282**
b7 Naturalness	0.224**
b8 Distinctiveness	0.395**
b9 Fascination	0.468**
b10 Landscape beauty	0.396**

*Correlation significant at the 0.05 level (two-tailed); **Correlation is significant at the 0.01 level (two-tailed).

#### The correlation between landscape preference related factors and evaluation of landscape change in present period

According to the findings presented in Table 10, it is evident that the research hypotheses H2-b hold true, as there exists a notable correlation between evaluation of landscape change and landscape preference related factors in the present period. The only factor exhibiting a negative correlation was disturbance, whereas complexity, coherence, mystery, stewardship, openness, naturalness, distinctiveness, fascination, and landscape beauty displayed positive correlations.

**Table 10:** The correlation between evaluation of landscape change and landscape preference related factors in the present period.

Landscape preference related factors	Evaluation of landscape change
a1 Complexity	0.219**
a2 Coherence	0.586**
a3 Mystery	0.470**
a4 Stewardship	0.540**
a5 Disturbance	− 0.457**
a6 Openness	0.394**
a7 Naturalness	0.439**
a8 Distinctiveness	0.593**
a9 Fascination	0.614**
a10 Landscape beauty	0.639**

*Correlation significant at the 0.05 level (two-tailed); **Correlation is significant at the 0.01 level (two-tailed).

### Analysis of linear relationship between landscape preference related factors and evaluation of landscape change

#### Linear relationship between landscape preference related factors and evaluation of landscape change in the classic farming and fishing villages period

According to Table 11, it is evident that there is a significant linear relationship between the evaluation of landscape change and the dimensions of fascination and disturbance in the period of Classic Farming and Fishing Villages, supporting research hypothesis H3-a. A regression model was constructed in the following manner: evaluation of landscape change during the Classic Farming and Fishing Villages period = 0.343* Fascination − 0.239* Disturbance, with an associated explanatory power of 0.251.

**Table 11:** Linear relationship between landscape preference related factors and evaluation of landscape change in the classic farming and fishing villages period.

	Non-standardised coefficients		Standardised coefficients	T	Sig	Adjusted R^2^	Common linear statistics	
	B	Standard error	β				Allowance	Standard error
(Constant)	5.622	1.026		5.482	0.010	0.251		
Fascination	0.781	0.190	0.343	4.110	0.000		0.730	1.370
Disturbance	− 0.611	0.213	− 0.239	− 2.865	0.005		0.730	1.370

Durbin–Watson 2.010; K–S check value 0.606.

#### Linear relationships between landscape preference related factors and evaluation of landscape change in the present period

As indicated in Table 12, research hypothesis H3-b is confirmed. A notable linear correlation exists between evaluation of landscape change in the present and the attributes of landscape beauty, stewardship, and coherence. Establish a regression model as: evaluation of landscape change in the present period = 0.415* Landscape beauty + 0.181* Stewardship + 0.194* Coherence. The model’s capacity for explanation is measured at 0.466. This model meets the criteria for normality, constancy, and independence checks, and does not exhibit any issues with covariance. Furthermore, the model illustrates that landscape aesthetics, stewardship, and coherence exert a favorable influence. In this context, the most significant factor is landscape beauty, followed by coherence, with stewardship being the least influential.

**Table 12:** Linear relationships between landscape preference related factors and evaluation of landscape change in the present period.

	Non-standardised coefficients		Standardised coefficients	T	Sig	Adjusted R^2^	Common linear statistics	
	B	Standard error	β				Allowance	Standard error
(Constant)	0.667	0.604		1.103	0.272	0.466		
Landscape beauty	0.946	0.183	0.415	5.185	0.000		0.566	1.766
Stewardship	0.368	0.171	0.181	2.154	0.033		0.512	1.953
Coherence	0.395	0.188	0.194	2.095	0.038		0.424	2.358

Durbin–Watson 1.665; K–S check value 0.246.

### The relationship between landscape ecological index, landscape preference related factors, and evaluation of landscape change

**Fig. 5:**
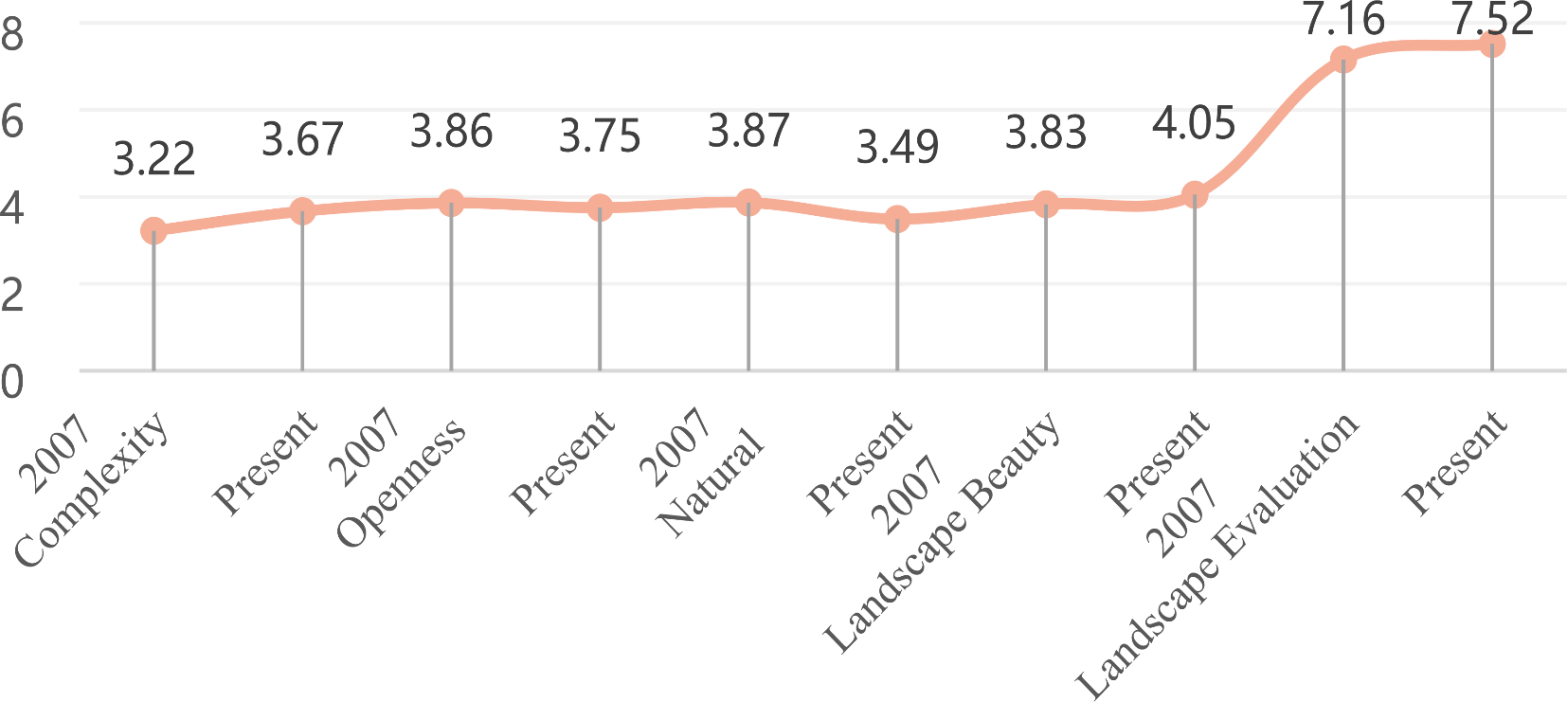
Comparative analysis chart of landscape preference related factors between the Classic Farming and Fishing Village in 2007 and the present period.

**Fig. 6:**
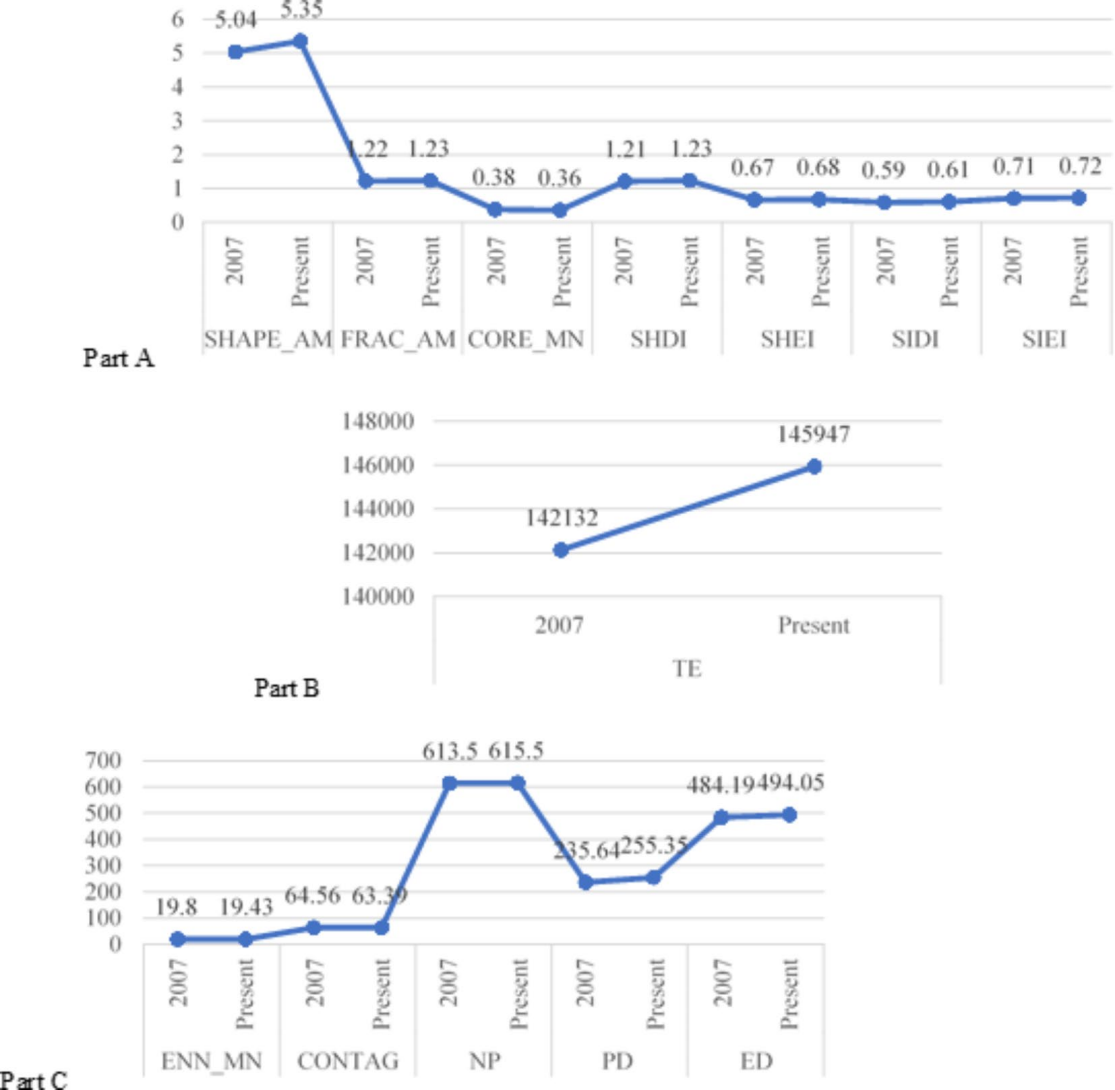
Comparative analysis chart of landscape ecological indicators between the Classic Farming and Fishing Villages in 2007 and the present period.

The literature revealed positive correlations between landscape complexity, landscape beauty, and evaluation of landscape change with SHAPE_AM, FRAC_AM, SHDI, SHEI, SIDI, SIEI, TE, NP, PD, and ED, while showing negative correlations with Core_MN, ENN_MN, and CONTAG. Figure 5 illustrates the shifts in landscape preference related factors and the evaluation of landscape change within rural communities across two distinct periods. Specifically, complexity, landscape beauty, and overall landscape evaluation scores exhibited increases from 3.22 to 3.67, 3.83 to 4.05, and 7.16 to 7.52, respectively. According to Fig. 6, the present period shows increased values compared to those of 2007 for SHAPE_AM, FRAC_AM, SHDI, SHEI, SIDI, SIEI, TE, NP, PD, and ED. Conversely, Core_MN, ENN_MN, and CONTAG exhibit lower values during the present period relative to 2007.

The degree of openness and naturalness demonstrates a positive association with Core_MN, ENN_MN, and CONTAG, while exhibiting a negative correlation with SHAPE_AM, FRAC_AM, SHDI, SHEI, SIDI, SIEI, TE, NP, PD, and ED. Figure 5 illustrates landscape preference related factors and evaluation of landscape change scores of rural communities during the two periods. It is observed that the openness scores decreased from 3.86 to 3.75, while the naturalness scores increased from 3.87 to 3.49. According to Fig. 6, Core_MN, ENN_MN, and CONTAG exhibit decreased values compared to those in 2007, while SHAPE_AM, FRAC_AM, SHDI, SHEI, SIDI, SIEI, TE, NP, PD, and ED (Fig. 6) demonstrate higher values at present than in 2007.

## Discussion

### Differences in landscape preferences related factors of rural communities in different periods

The results of the study showed that there were significant differences in landscape preference related factors across time in rural communities, with a greater preference for the naturalness of the Classic Farming and Fishing Villages period. Complexity, coherence, mystery, stewardship, distinctiveness, fascination, and landscape beauty in the present period are higher than in the Classic Farming and Fishing Villages period. Based on the results obtained thus far, it is evident that Taiwan’s rural development policy is appropriate and largely aligns with the landscape preferences of rural residents. Over more than five decades of development in Taiwan’s rural communities, the policy has evolved into a synthesis of government-led, top-down strategies and public-initiated, bottom-up approaches. Prior to 2000, rural policies predominantly focused on government-led initiatives aimed at enhancing agricultural production and improving rural livelihoods^65^. The rural policy, from 2000 to the present, has been guided by the government and promotes the synergistic development of life, culture, production, and ecology^65^. Moreover, there is a strong emphasis on the bottom-up approach and respect for local autonomy. The Ministry of Agriculture^66^ established the "Plan for Creating a New Landscape for Rural Areas", scheduled for implementation from 2000 to 2008. The project focuses on redeveloping rural settlements, improving the rural living environment, constructing new residential areas, and shaping a new rural landscape. The project is dedicated to enhancing rural economic strength, improving the working and living conditions of villagers, reducing the urban–rural gap, and establishing a new, modern rural living area. Moreover, the program aims to create a prosperous countryside by harmonizing agricultural production, farmers’ lives, and the rural natural ecology. The Soil and Water Conservation Bureau organized a competition to identify the top ten Classic Fishing and Farming villages^40^, significantly enhancing their development. This event marks the inception of the timeline for this study. The Soil and Water Conservation Bureau^67^ developed the "Revitalisation of the Economy and Expansion of Public Construction Investment Plan". This plan primarily encompasses the following objectives: the preliminary planning for rural regeneration construction, the participatory greening of rural living environments, and the expedited enhancement of rural infrastructure. The Soil and Water Conservation Bureau^68^ enacted the "Rural Regeneration Ordinance". This ordinance aims to enhance the quality of life for rural populations, generate employment opportunities, increase the income of rural residents, and improve the overall rural environment. The implementation strategy is characterized by a bottom-up, project-oriented approach that emphasizes community self-governance and integrates both hard and soft infrastructure development, with the overarching goal of fostering vibrant, healthy, and thriving rural communities. The Rural Regeneration Programme is structured into three distinct phases: the initial phase spans from 2012 to 2015^69^; the second phase extends from 2016 to 2019^70^; and the third phase covers the period from 2020 to 2023^71^. Notably, the third phase also marks the conclusion of the study’s temporal framework. These policies can serve as references for rural development initiatives in other countries and regions, facilitating the achievement of environmental sustainability and the enhancement of human well-being. Nonetheless, it is imperative to prioritize the natural integrity of private land, implement appropriate incentive and subsidy measures for private landowners, and enhance regulatory oversight to ensure the preservation and high quality of these lands. Effective management of publicly-owned land involves rational spatial allocation, rigorous vetting and approval processes, and ensuring that ecological and agricultural land statuses are not subject to arbitrary changes. Furthermore, future policy formulation can leverage existing policies, placing greater emphasis on fostering and enhancing the internal dynamics of rural areas while promoting sustainable rural development.

### The correlation between landscape preference related factors and evaluation of landscape change

(1) The landscape preference related factors during the two periods show a correlation with the evaluation of landscape change. The relevant factors are coherence, mystery, stewardship, openness, naturalness, distinctiveness, fascination, and landscape beauty. Disturbance exhibits a negative correlation with it, whereas all other factors demonstrate positive correlations. The outcomes of this study align closely with the research discoveries presented by Ning and Ou^72^. During the period of Classic Farming and Fishing Villages, complexity held no significance, whereas it bears relevance in the present period. While Ulrich^73^ research did not observe a correlation between respondents’ preference evaluation and complexity, subsequent studies have identified such a relationship. Herzog and Kropscott^74^ discovered a clear association between participants’ assessments of complexity and their evaluations of preference. Van der Jagt et al.^75^ discovered a moderate positive association between complexity and the evaluation of landscape change. Tang et al.^76^ identified a clear correlation between preferences for complexity in descriptions of forest interiors, forest edges, and scenes of deforestation. The complexity of a landscape profoundly influences its visual appeal^77^. There exists a notable positive correlation between the evaluation of landscape change and coherence, aligning with observations reported by previous researchers^74,75,77^. In a meta-analysis encompassing findings from 11 studies, Stamps^78^ identified coherence as exhibiting the strongest correlation with evaluation of landscape change. Van der Jagt et al.^75^ confirmed the validity of the Landscape Preference Matrix, revealing that coherence positively influences preferences for landscape beauty. The coherence of a landscape significantly influences its visual appeal, as landscapes lacking coherence are generally less preferred, whereas images displaying medium to high coherence are favored^79–81^. Herzog and Kropscott^74^ discovered a favorable association between mystery and evaluation of landscape change. Gifford^82^ suggests that there is a preference for scenes imbued with a mystical quality, with natural scenes perceived as more realistic compared to alternative settings. In Van den Berg and Koole^83^ investigation of rural landscapes in Denmark, it was demonstrated that stewardship has a beneficial impact on the evaluation of landscape change. Specifically, the study found that residents in the area exhibit a stronger preference for landscapes that are actively stewardship. In their investigation of rural villages in eastern China, Peng et al.^6^ discovered that favorable assessments of enhancements in rural land management played a crucial role in bolstering residents’ favorable attitudes towards changes in the landscape. Stewardship pertains to the management of natural resources and the safeguarding of the environment aimed at preserving favorable landscape characteristics^84,85^. Sharafatmandrad and Khosravi Mashizi^77^ investigated the potential adverse effects of contemporary mismanagement on the aesthetic appeal of rangeland landscapes. Daniel^48^ and Gobster et al.^54^ contend that naturalness plays a crucial role in shaping individuals’ preferences. Van den Born et al.^86^ examined the evaluations of Dutch inhabitants regarding alterations in river landscapes following significant projects. They discovered that residents show a preference for environments characterized by a strong sense of place identity and natural elements. The visual appeal of the landscape has the potential to enhance the comfort and psychological well-being of residents^87^. The aesthetic qualities of a landscape impact the overall well-being of the public by influencing the pleasure and contentment experienced while engaging with the surroundings^20,87,88^. Sharafatmandrad and Khosravi Mashizi^77^ findings suggest that human interventions such as overgrazing, deforestation, and burning activities result in diminished vegetation cover, subsequently altering both the structure and functionality of landscapes.

(2) The evaluation of landscape change during the period of Classic Farming and Fishing Villages revealed a notable correlation between fascination and disturbance, following a linear trend. Fascination yielded favorable outcomes, whereas disturbance led to adverse effects. A notable linear correlation exists between the evaluation of landscape change at present and the elements of landscape aesthetics, stewardship, and coherence. The findings of this investigation align closely with those of previous research endeavors. Van der Jagt et al.^75^ contend that there exists a positive predictive relationship between landscape beauty and the factors of complexity and naturalness. Sahraoui et al.^89^ observed a positive correlation between the extent of landscape openness and human aesthetic evaluation, highlighting the significant role of farmland in shaping aesthetic values. Zhang et al.^90^ discovered a positive correlation between coherence and both stewardship and openness, while noting a negative correlation with richness. Furthermore, they observed that complexity was positively influenced by the depth of richness but negatively impacted by stewardship and openness. Ning and Ou^71^ discovered that landscape satisfaction was positively influenced by factors such as the landscape beauty, naturalness, and complexity of the environment. Sowińska-Świerkosz and Soszyński^91^ employed a selection of topographic features (T), water presence (W), vegetation percentage (PV), building percentage (PB), positive man-made elements (PE), negative man-made elements (NE), naturalness degree (DN), diversity (D), and openness (O) as components of the rural landscape preference prediction index. Notably, the percentage of buildings (PB), negative man-made elements (NE), and naturalness degree (DN) exerted a more pronounced influence within this index. This research proposes that as individuals experience improvements in their quality of life and develop a greater ecological consciousness over time, factors such as landscape beauty, stewardship, coherence, naturalness, and complexity become increasingly pivotal in evaluating landscape preferences.

In summary, the findings above validate and extend Kaplan and Kaplan’s^51^ four landscape preference matrices, Tveit et al.'s^52^ nine landscape visual characterization frameworks, Hunter and Askarinejad’s^53^ incorporation of landscape-related theories to construct ten factors, and Ning and Ou’s^18^ qualitative research, which validated and extended the ten-factor study across three categories."Over time, facilitated by both formal and informal learning processes, there has been a demonstrated increase in tolerance towards architectural and landscape impacts influenced by human behavior^92^, thereby fostering greater acceptance of sustainable changes within landscapes^59^. Zhang et al.^93^ identified that rural inhabitants exhibited a preference for landscapes characterized by a blend of traditional rural elements alongside modern attributes. Moreover, their interaction with urban areas heightened their inclination towards rural amenities and landscape characteristics. Effectively managing the environment constitutes a protracted endeavor that imposes heightened requirements on urban planners, land stewards, and governmental entities. The findings of this study can serve as a valuable reference for the prospective advancement of rural environments. This includes leveraging the lessons from the transformative and developmental significance of village landscapes, thereby fostering a multidimensional approach. This approach enables the management of landscape coherence, mystery, stewardship, openness, naturalness, distinctiveness, fascination, and landscape beauty.

### Relationship of landscape ecological indicators with landscape preferences related factors and evaluation of landscape change

By linking objective and subjective data, this study reveals the relevance of landscape ecological indicators to landscape preference and the evaluation of landscape change, and its findings are both compatible with existing studies and have new findings. In rural areas, the shape indices, diversity indices, edge indices, and density indices of landscape ecological indicators and the complexity, landscape beauty, and landscape change evaluations of landscape preferences related factors have the potential to be raised and lowered together. The results of this study have similarities with other studies^62–64,94,95^, which have pointed out that the diversity indices (SIEI, SHDI, and SIDI) and shape indices (SHAPE_AM and FRAC_AM) show a positive correlation with landscape beauty. The contagion index (CONTAG) showed a negative correlation with landscape beauty, which is similar to the findings of Schirpke et al.^63^. The diversity index (SHDI) and density index (NP and PD) were positively correlated with landscape preference, which is similar to the findings of Dramstad et al.^15^ and Kuper^95^. The Edge Index (ED) is positively correlated with the evaluation of landscape change, which is similar to the findings of Palmer^24^. The core area, proximity, and sprawl indices of landscape ecological indicators and the complexity, landscape beauty, and landscape change evaluations of landscape preferences have the potential to conflict with each other. The results may provide new insights into the relationship between landscape ecological indicators and landscape preferences.

Numerous studies have confirmed that the natural environment is closely related to human health and well-being^96^. Particularly in rural communities, the ecological and visual quality of the landscape often directly affects the quality of life of residents^96^. Then, it is particularly important to understand the relationship between landscape ecological indicators and people’s landscape preferences and evaluation of landscape changes in rural communities^61^. Understanding the relationship between visual landscape quality and ecological attributes is a key issue that must be explicitly considered in landscape management and ecological conservation programmes^97^. The results of the study show that there is a correlation between landscape ecological indicators and landscape preferences. The ultimate goal of landscape planning and design in rural communities is to enhance ecological quality while also satisfying people’s landscape preferences. In future rural landscape planning and design, the scientific and rational allocation and utilization of land will be crucial. It is crucial to not only understand the spatial distribution of ecological quality and characteristic areas in rural communities, but also to examine the spatial patterns and attributes of landscapes preferred by residents. Ultimately, design serves to harmonize ecological quality with landscape preferences, thereby promoting human health and well-being.

### Research limitations

There are few limitations in this study. The limitation of this study is the lack of quantitative verification of the relationship between landscape ecological indicators and landscape preferences. The landscape ecological indicators were not calculated at multiple scales, excluding visual invisibility. The landscape ecology index is limited to the landscape index and more indicators should be included in the discussion. In future studies, a quantitative link between landscape ecological indicators and landscape preferences could be established. The calculation of landscape ecological indicators takes into account multi-scale and visual visibility factors. At the same time, more landscape ecological indicators can be included to increase the sample size for a mixed study design so that the results can be further validated.

## Conclusions

In the realm of alterations to the landscape environment, noticeable variations exist in the preferences for landscape characteristics (such as complexity, coherence, stewardship, naturalness, fascination, and landscape beauty) among rural communities across the two distinct periods. The factors influencing landscape preferences are closely correlated with the evaluation of landscape changes. In the present period, there exists a notable linear correlation between the evaluation of landscape changes and the attributes of landscape beauty, stewardship, and coherence. Landscape ecological indicators exhibit associations with factors such as landscape complexity, landscape beauty, openness, naturalness, and the evaluation of landscape changes. The study reveals that human cognitive abilities are heightened when encountering dynamic landscape environments. The shifting landscape environment fosters enhancements in human cognition. The findings of this research reflect the pertinent concerns of rural community residents, offering valuable insights to shape and execute landscape policies within these areas. When planning and designing rural community landscapes in the future, in addition to paying attention to the naturalness of the landscape, it also needs to develop collaboratively with landscape beauty, stewardship, coherence, openness, and uniqueness to satisfy the residents’ preference evaluation of the landscape changes. In forthcoming studies, it would be advantageous to employ quantitative research to further substantiate the correlation between landscape ecological indicators, preferences for landscape, and the evaluation of landscape changes.

## Data Availability

All data generated or analysed during this study are included in this published article.
